# Supplementary material for: SyNDI: synchronous network data integration framework
Source: BMC Bioinformatics. 2018 Nov 6;19:403. doi: 10.1186/s12859-018-2426-5 (PMC6219086; doi:10.1186/s12859-018-2426-5)
Supplement: Supplementary file 5 — Files of the example presented in Additional file 4. This additional file is a zip package that contains all files used in Additional file 4. Detailed descriptions of each of these files are presented in a README file included in this zip package. (GZ 30411 kb) [file 12859_2018_2426_MOESM5_ESM.gz › SyNDITools/Meme2Fimo/meme_4.9.0_4.tar.gz!/meme_4.9.0/doc/examples/mcast_example_output_files.tgz!/mcast_example_output_files/mcast.html]

MCAST


For further information on how to interpret these results or to get a
copy of the MEME software please access
http://meme.nbcr.net.

Inputs
  |  
Search Results
  |  
Program information
  |  
Documentation

|  |  |
| --- | --- |
| Inputs | Top |

#### Sequence Database

The sequence database
"crp0.s"
was supplied to MCAST.

| Sequence Count | Residue Count |
| --- | --- |
| 18 | 1890 |

#### Motifs

The following motifs were supplied to MCAST from
"crp0.meme.html"

|  |  |  |  |
| --- | --- | --- | --- |
| Motif | Width | Best possible match | Best possible RC match |
| 1 | 18 | AATTGTGACGTTGTTCAC | GTGATCTACGTCACAATT |
| 2 | 8 | CGGTGGGG | CCCCGCCG |
| 3 | 11 | CCTGGTGTGCC | GGGACCCCAGG |

|  |  |
| --- | --- |
| Search Results | Top |

#### Top Scoring Matches

Each of the following
17 matches
has a *q*-value less than
.
  
The motif matches shown have a position p-value less than
.  
**Click on the arrow** (↧) next to the block diagram to view more information about a sequence.

|  |  |
| --- | --- |
|  | Motif 1 |

|  |  |
| --- | --- |
|  | Motif 2 |

|  |  |
| --- | --- |
|  | Motif 3 |

| Sequence | Start | Stop | Score | *p*-value | *E*-value | *q*-value |  |  |
| --- | --- | --- | --- | --- | --- | --- | --- | --- |
| lac | 9 | 44 | 17.8792 | -- | -- | -- |  |  |
| +  - | | | | | | | | ↧ |
| trn9cat | 36 | 102 | 10.9305 | -- | -- | -- |  |  |
| +  - | | | | | | | | ↧ |
| ilv | 5 | 61 | 9.60584 | -- | -- | -- |  |  |
| +  - | | | | | | | | ↧ |
| ara | 59 | 77 | 7.17504 | -- | -- | -- |  |  |
| +  - | | | | | | | | ↧ |
| male | 14 | 49 | 6.74549 | -- | -- | -- |  |  |
| +  - | | | | | | | | ↧ |
| malt | 41 | 59 | 6.51379 | -- | -- | -- |  |  |
| +  - | | | | | | | | ↧ |
| deop2 | 60 | 78 | 5.24387 | -- | -- | -- |  |  |
| +  - | | | | | | | | ↧ |
| pbr322 | 57 | 75 | 4.32247 | -- | -- | -- |  |  |
| +  - | | | | | | | | ↧ |
| uxu1 | 17 | 35 | 4.08176 | -- | -- | -- |  |  |
| +  - | | | | | | | | ↧ |
| ce1cg | 65 | 83 | 3.76129 | -- | -- | -- |  |  |
| +  - | | | | | | | | ↧ |
| tnaa | 75 | 93 | 2.964 | -- | -- | -- |  |  |
| +  - | | | | | | | | ↧ |
| gale | 46 | 64 | 2.964 | -- | -- | -- |  |  |
| +  - | | | | | | | | ↧ |
| crp | 67 | 85 | 2.46002 | -- | -- | -- |  |  |
| +  - | | | | | | | | ↧ |
| cya | 50 | 68 | 2.2681 | -- | -- | -- |  |  |
| +  - | | | | | | | | ↧ |
| ompa | 48 | 66 | 1.79464 | -- | -- | -- |  |  |
| +  - | | | | | | | | ↧ |
| tdc | 82 | 100 | 1.73904 | -- | -- | -- |  |  |
| +  - | | | | | | | | ↧ |
| malk | 61 | 79 | 1.5644 | -- | -- | -- |  |  |
| +  - | | | | | | | | ↧ |

Top

##### MCAST version

4.8.0 (Release date: 7 EST 20)

##### Reference

Timothy Bailey and William Stafford Noble,  
"Searching for statistically significant regulatory modules",
*Bioinformatics (Proceedings of the European Conference on Computational Biology)*,
19(Suppl. 2):ii16-ii25, 2003.

##### Command line summary

mcast crp0.meme.html crp0.s  
Background letter frequencies (from non-redundant database):  

A: 0.282   C: 0.222   G: 0.229   T: 0.267

show model parameters...

hide model parameters...

|  |  |
| --- | --- |
| Explanation of MCAST Results | Top |

#### The MCAST results consist of

- The **inputs** to MCAST including:
  1. The **sequence database** showing the sequence
     and residue counts. [View]
  2. The **motifs** showing the name, width,
     and best scoring match [View]
- The **search results** showing top scoring sequences with
  tiling of all of the motifs matches shown for each of the sequences. [View]
- The **program** details including:
  1. The **version** of MCAST and the date it was released. [View]
  2. The **reference** to cite if you use MCAST in your research. [View]
  3. The **command line summary** detailing the parameters with which you ran MCAST. [View]
- This **explanation** of how to interpret MCAST results.

#### Inputs

MCAST received the following inputs.

##### Sequence Databases

This table summarises the sequence databases specified to MAST.

Database
:   The name of the database file.

Sequence Count
:   The number of sequences in the database.

Residue Count
:   The number of residues in the database.

##### Motifs

Summary of the motifs specified to MAST.

Name
:   The name of the motif. If the motif has been removed or removal is recommended to avoid highly similar motifs
    then it will be displayed in red text.

Width
:   The width of the motif. No gaps are allowed in motifs supplied to MAST as it only works for motifs of a fixed width.

Best possible match
:   The sequence that would achieve the best possible match score and its reverse complement for nucleotide motifs.

#### Search Results

MCAST provides the following motif search results.

##### Top Scoring Sequences

This table summarises the top scoring sequences.
The sequences are sorted by the Sequence
*q*-value from most to least significant.

Sequence
:   The name of the sequence. This maybe be linked to search a sequence database for the sequence name.

Start
:   The position of the start of the match in 1-based coordinates, starting at the
    first position in the sequence.

Stop
:   The position of the end of the match in 1-based coordinates, starting at the
    first position in the sequence.

Score
:   The MCAST score for the match.

*p*-value
:   The *p*-value of the match score.

*E*-value
:   The *E*-value of the match score.

*q*-value
:   The *q*-value of the match score.

↧
:   Click on this to show additional information about the sequence such as a
    description, combined p-value and the annotated sequence.

Block Diagram
:   The block diagram shows the best non-overlapping tiling of motif matches on the sequence.

    - The length of the line shows the length of a sequence relative to all the other sequences.
    - A block is shown where the positional *p*-value
      of a motif is less (more significant) than the significance threshold which is 0.0001 by default.
    - If a significant motif match (as specified above) overlaps other significant motif matches then
      it is only displayed as a block if its positional *p*-value
      is less (more significant) then the product of the positional
      *p*-values of the significant matches that it overlaps.
    - The position of a block shows where a motif has matched the sequence.
    - The width of a block shows the width of the motif relative to the length of the sequence.
    - The colour and border of a block identifies the matching motif as in the legend.
    - The height of a block gives an indication of the significance of the match as
      taller blocks are more significant. The height is calculated to be proportional
      to the negative logarithm of the positional *p*-value,
      truncated at the height for a *p*-value of 1e-10.
    - Hovering the mouse cursor over the block causes the display of the motif name
      and other details in the hovering text.
    - DNA only; blocks displayed above the line are a match on the given DNA, whereas blocks
      displayed below the line are matches to the reverse-complement of the given DNA.
    - DNA only; when strands are scored separately then blocks may overlap on opposing strands.


+ 

  
  
  
  
  
  
  
  
  
  
  
  
  
  
  
  
  
  
  
  
  
  
  
  
  
  
  
  
  
  
  
  
  
  
  
  
  
  
  
  
  
  
  
  
  
  
  
  
  
  
  
  
  
ACGTN
